# Evidence in hypertensive rats of hypotensive effect after mandibular extension

**DOI:** 10.14814/phy2.13911

**Published:** 2018-12-13

**Authors:** Cristina Del Seppia, Dominga Lapi, Sergio Ghione, Giuseppe Federighi, Laura Sabatino, Enza Fommei, Antonio Colantuoni, Rossana Scuri

**Affiliations:** ^1^ Institute of Clinical Physiology National Council of Research (CNR) Pisa Italy; ^2^ Department of Clinical Medicine and Surgery “Federico II” University Medical School Naples Italy; ^3^ Fondazione Toscana Gabriele Monasterio ‐ Medical and Public Health Research Pisa Italy; ^4^ Department of Translational Research on New Technologies in Medicine and Surgery University of Pisa Pisa Italy

**Keywords:** Blood pressure, heart rate, hypertension, mandibular extension, rats

## Abstract

Previous studies in anesthetized normotensive rats demonstrated that a single mouth opening for 10 min obtained by an ad hoc dilator (mandibular extension [ME]) produced a blood pressure reduction by about 20 mmHg lasting for about 2 h and that once‐repeated ME prolonged this effect. We here describe these effects in hypertensive rats. Mean (intra) arterial blood pressure (MABP) and heart rate (HR) was followed for up to a maximum of 470 min after single or repeated 10 min‐lasting ME in two groups of anesthetized, male, 6–9 months old hypertensive rats. In one group, hypertension was induced by dexamethasone (20 *μ*g/kg/day, subcutaneously for 7 days; Dex‐HT); the other group was spontaneously hypertensive rats (SHR). Studies were done, in Dex‐HT rats, after only surgical procedures (no ME, sham‐operated rats), single ME, early repeated (after 10 min) ME (ER‐ME) and late repeated (after 160 min) ME (LR‐ME) and, in SHR, after only surgical procedures and ER‐ME. One‐way ANOVA for repeated measures revealed no significant effect on MABP and HR in sham‐operated groups. In Dex‐HT rats, single ME was followed by a significant MABP decline by 25 mmHg, lasting for 100 min; ER‐ME and LR‐ME were followed by an even greater significant MABP decline by 40 mmHg, which outlasted the experimental observation period. In SHR, ER‐ME gave similar results as in Dex‐HT rats. HR significantly declined in all, except sham‐operated groups. In conclusions, ME is followed by a prolonged MABP decline also in hypertensive rats. This effect is even more pronounced, in length and magnitude, after repeated ME.

## Introduction

Several evidence indicate that manipulations in the facial region can induce autonomic reflexes that result in blood pressure (BP) and heart rate (HR) reduction in both animals (Kumada et al. 1977) and humans (Cornelius et al. 2010; Schaller et al. 2009). In recent years, we have described a hypotensive and bradycardic effect after a mouth opening, obtained by an ad hoc device, in both normotensive anesthetized rats and in normotensive humans (Lapi et al. 2013, 2014, 2017; Del Seppia et al. 2016, 2017). We defined this mouth opening as mandibular extension (ME). Ten minutes ME produced effects on BP and HR similar but of different entity in the rat respect to humans. In fact, a lasting (up to 140 min) decrease of about 20 mmHg of mean blood pressure (MBP) was detected in the rat (Lapi et al. 2013, 2014), while normotensive volunteers showed a prolonged systolic and diastolic pressure reduction of lesser extent (about 3–4 mmHg) when subjected to two different dilator devices, one allowing partial masticatory movements, defined dynamic extension (Brunelli et al. 2012; Del Seppia et al. 2016), and another with a fixed mouth opener, defined static extension (Del Seppia et al. 2017), however, a similar study by other authors using dynamic extension failed to confirm these results (De Innocentiis et al. 2015).

More recently, we reported that, in normotensive rats, the application of a second ME 10 min after the first one was followed by a hypotensive effect that was of similar magnitude as a single ME, but was much more prolonged since it extended over the entire experimental observation period of 240 min (Lapi et al. 2017).

The aim of the present paper was to extend these findings by performing studies on the effects of ME repetition on blood pressure and heart rate in hypertensive rats. The major part of the studies was done in dexamethasone‐induced hypertensive (Dex‐HT) rats. In addition, a few exploratory studies were also done in spontaneously hypertensive rats (SHR).

## Material and Methods

### Animals and housing

Twenty‐five adult males Wistar rats (250–300 g), from Harlan (Udine, Italy) and ten male spontaneously hypertensive rats (SHR) (250–350 g), from Charles River (Calco, SO, Italy) were used. DEX‐HT was induced in Wistar rats by subcutaneous daily administration of dexamethasone (20 *μ*g/kg/day) for 7 days.

All rats were 6–9 months old and were housed in polyethylene cages, under a 12/12 h light/dark cycle (light 8:00–20:00 h) at constant temperature (24 ± 1°C) and humidity (60 ± 5%) with free access to food and water.

This study was carried out in accordance with the recommendations in the Guide for the Care and Use of Laboratory Animals of the National Institute of Health. The protocol was approved by the Committee on the Ethics of Animal Experiments of the University of Pisa and Ministry of Health (Permit Number: 157/2017‐PR). All surgery was performed under alpha‐chloralose and urethane anesthesia and all efforts were made to minimize suffering.

### Surgical procedure

During the whole experiments, the rats were anesthetized. Anesthesia was induced with an intraperitoneal injection of alpha‐chloralose (50 mg/Kg b.w.) (Sigma‐Aldrich, St. Louis, MO) plus urethane (600 mg/Kg b.w.) (Sigma‐Aldrich) and maintained with following administration of urethane alone (100 mg/kg, i.v., every hour) through a catheter placed in the right femoral vein. As previously described (Lapi et al. 2013, 2014, 2017), rats were tracheotomized, intubated and mechanically ventilated with room air, and a supplemental mixture of O_2_/CO_2_ (end‐tidal CO_2_ was continuously measured by a CO_2_ analyzer and a respirator was adjusted to maintain end‐tidal CO_2_ from 4.5% to 5.0% and to keep arterial blood gas tension within the normal range).

A polyethylene cannula was placed in the left femoral artery to measure Mean (intra) arterial blood pressure (MABP).

The rats were secured to a heating stereotaxic frame which allowed to maintain the body temperature at 37.0 ± 0.5°C measured with a rectal probe.

### Blood pressure and heart rate measurements

Mean arterial blood pressure was monitored with a blood pressure transducer model BLPR2 (World Precision Instruments, Sarasota, FL) in contact with the catheter placed in the left femoral artery, connected to an ad hoc bridge amplifier (home‐made) and recorded using LabView software (National Instruments S.R.L., Milan, Italy), which measured MABP values every 60 sec, automatically providing an averaged value every 5 min.

**Figure 1:**
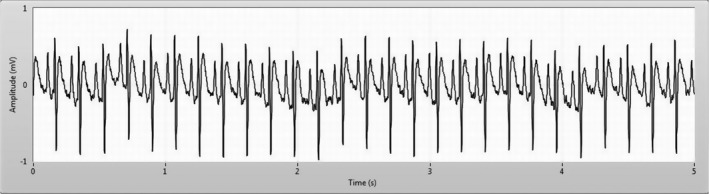
Example of ECG trace recorded in normotensive rat in baseline conditions (I derivation).

ECG recordings (at a sampling rate of 1 KHz) were performed applying the electrodes under the skin according to the three derivations of Einthoven, as is commonly done in anesthetized rats (Konopelski and Ufnal 2016) (Fig. 1). The signals were captured with a home‐made differential amplifier for biomedical signals and data acquisition was obtained by LabView software. HR measurements were done on 5 sec acquisitions according to the following procedure: (1) the signal was filtered with a band pass filter (cut‐off frequencies 5–40 Hz), (2) the value of the mean and the standard deviation of the filtered signal over the 5 sec interval were determined (3) R peaks were recognized considering the mean + 1.5 standard deviation (SD) as threshold of the signal and the sign changes of the derivative (4) HR was obtained by calculating the number of *R* peaks in the time unit (1 min). Values at intervals of 10 and 20 min were used for statistical analysis and for graphical representation. For the baseline values, the averages of three recordings obtained at 10 min intervals immediately prior to the first ME were used. For the first 30 min post‐ME1 period, recordings at 10 min interval were used. For the subsequent observation period, recordings at 20 min interval were used.

Values at intervals of 10 and 20 min were used for statistical analysis and for graphical representation. For the baseline values, the average of three recordings obtained at 10 min intervals immediately prior to the first ME were used. For the first 30 min posttreatment period, recordings at 10 min interval were used. For the subsequent observation period, recordings at 20 min interval were used.

### Mandibular extension

ME was induced by a home‐made U‐shaped dilator, appropriately designed for rats, that was placed between the superior and inferior dental arches of the animal. The dilator consisted of two thin layers covered with a silicone elastomer (Sylgard, Dow Corning, Midland, MI) coupled to an adjustable spring. The degree of ME was set at the largest opening of the mouth that did not produce any muscle fatigue, as previously assessed in pilot experiments in which various degrees of opening of the mouth was applied and the tension of the masseter and anterior temporal muscles was evaluated by electromyography (Lapi et al. 2013). All ME lasted for 10 min.

### Experimental procedure

#### Dex‐HT rats

The rats were randomly assigned to one of four groups: (1) sham‐operated rats; (2) rats submitted to one ME (defined as single ME rats); (3) rats submitted to two MEs separated by 10 min (early repeated ME rats), and (4) rats submitted to two MEs separated by 160 min (late repeated ME rats).

1. Sham‐operated rats (*n* = 5) were subjected to all surgical procedures (but not ME) and kept under observation for 300 min.
2. Single ME rats (*n* = 5) were kept under observation for 30 min (basal period), after which they were subjected to ME. The entire observation period was 300 min.
3. Early repeated ME rats (*n* = 10) were kept under observation for 30 min, after which they were subjected, like single ME rats, to a first ME (ME1), which was however followed by a second ME (ME2) after 10 min. The entire observation period was 300 min.
4. Late repeated ME rats (*n* = 5) were subjected to same protocol, as early repeated ME rats, with the difference that ME2 was repeated 160 min after ME1. The entire observation period was 470 min.

#### Spontaneously hypertensive rats

Two groups were studied: (1) sham‐operated rats (*n* = 4), which were subjected to surgical procedures, but not ME and kept under observation for 300 min and, (2) early repeated ME rats (*n* = 6), which underwent the same protocol applied to the early repeated ME DEX‐HT rats described in the previous paragraph.

### Statistical analysis

Blood pressure and heart rate data were summarized as mean ± SD and plotted as mean ± standard error of the mean (SEM). Statistical analysis was done using one‐way analysis of variance (ANOVA) for repeated measures. When the ANOVA revealed a statistically significant effect (*P* < 0.05), the Holm‐Sidak test was run for “post hoc” comparisons. All analyses were done with the statistical package Sigma Stat, version 3.5 (Jandel Corporation San Mateo, CA).

## Results

### Experimentally hypertensive rats (Dex‐HT rats)

**Figure 2:**
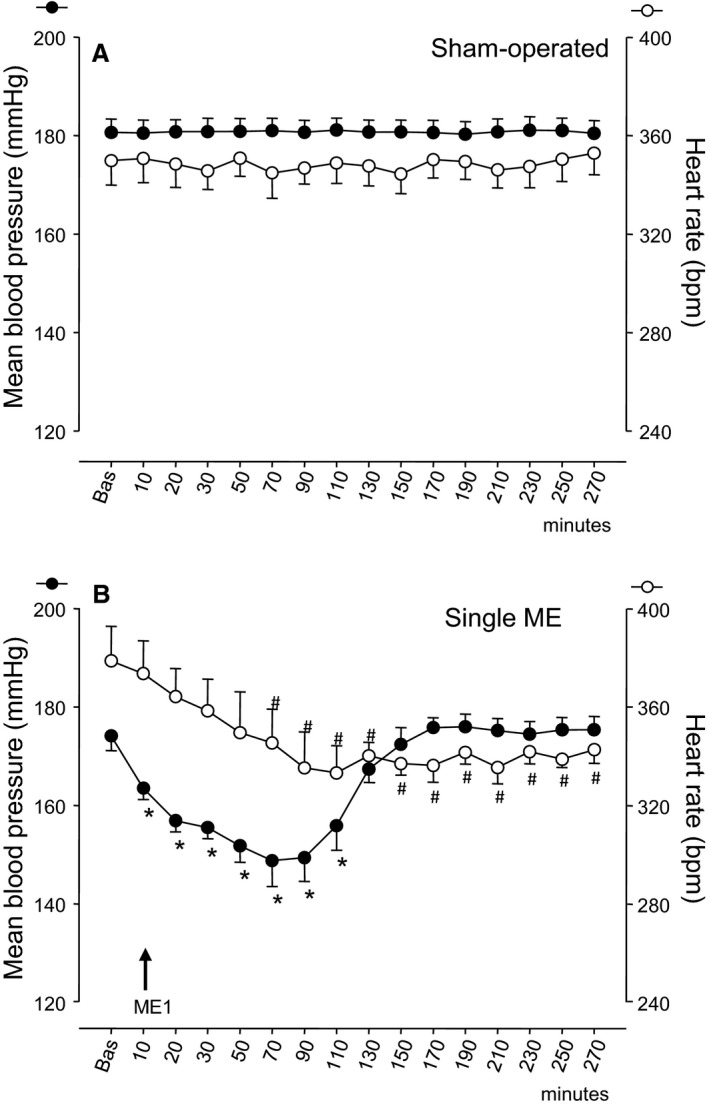
The time courses, in Dex‐HT rats, of the mean arterial blood pressure (left ordinate axis, filled circles) and heart rate (right ordinate axis, empty circles) in (A) sham‐operated rats and (B) single ME treated rats. ME with arrow indicates the timing of ME treatment, and “Bas” indicates the mean basal value. Values (means ± SEM) are plotted every 10 min for the first 30 min after basal value and every 20 min, thereafter. Asterisks (*) and hashes (#) indicate significant differences in post hoc comparisons between basal value and posttreatment values, respectively for mean blood pressure and heart rate. For other explanations see text.

**Figure 3:**
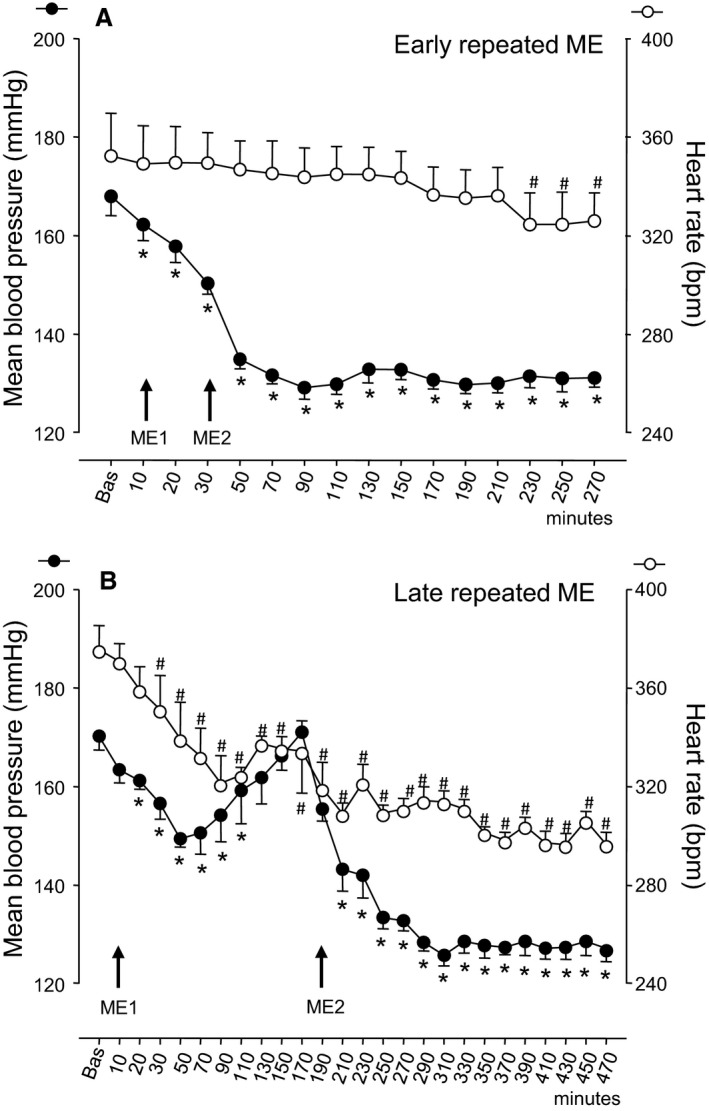
The time courses, in Dex‐HT rats, of the mean blood pressure (left ordinate axis, filled circles) and heart rate (right ordinate axis, empty circles) in (A) early repeated ME treated rats and (B) late repeated ME treated rats. ME1 and ME2 with arrows indicate the timing of ME treatments, and “Bas” indicates the mean basal value. Values (means ± SEM) are plotted every 10 min for the first 30 min after basal values and every 20 min, thereafter. Asterisks (*) and hashes (#) indicate significant differences in post hoc comparisons between basal value and posttreatment values, respectively for mean blood pressure and heart rate. For other explanations see text.

The time course of MABP and HR in the four study groups is reported in Figures 2 and 3. Post hoc comparisons are also reported, if significant.

For MABP, no significant change was present in the sham‐operated group for the entire observation period (Fig. 2A). In rats with a single ME (Fig. 2B), MABP started to diminish immediately after ME, declined by about 25 mmHg and attained a nadir about 1 h after ME (at 70 min in the Fig. 2B). Thereafter, it gradually increased attaining baseline values in the following hour and remaining then stable for the entire final observation period. Overall repeated measure ANOVA revealed a highly significant main effect (*P* < 0.001) and post hoc comparisons against the basline value were significant for the first 100 min.

In rats with early repeated ME (Fig. 3A), MABP started to diminish immediately after ME1, displaying the most marked decrease (of about 40 mmHg) 1 h after ME2 (at 90 min in the Fig. 3A) and remaining then stably low for the subsequent observation period of about 3 h. Overall repeated measure ANOVA revealed a highly significant main effect (*P* < 0.001) and post hoc comparisons against the baseline value were significant for all observation points after the first 10 min.

In rats with late repeated ME (Fig. 3B), the time course of MABP was identical to that of the single ME group for the first part of the study, that is, until ME2 was applied. Thereafter, MABP displayed a marked decrease of about 40 mmHg 120 min after ME2 and remained stably low for the entire final observation period of about 160 min. Overall repeated measure ANOVA revealed a highly significant main effect (*P* < 0.001) and post hoc comparisons against the baseline value were significant between 20 and 110 min, and then again for all observation points after 210 min.

The time course of HR is also reported in Figures 2 and 3. HR remained unchanged in the sham‐operated group and decreased significantly in the other three groups, in which the overall repeated measure ANOVA revealed a highly significant main effect of treatment (*P* < 0.001). Post hoc comparisons revealed significant differences from the baseline value from 70 min onwards in single ME rats, from 230 min onwards in early repeated ME rats and from 30 min onwards in late repeated ME.

### Spontaneously hypertensive rats

**Figure 4:**
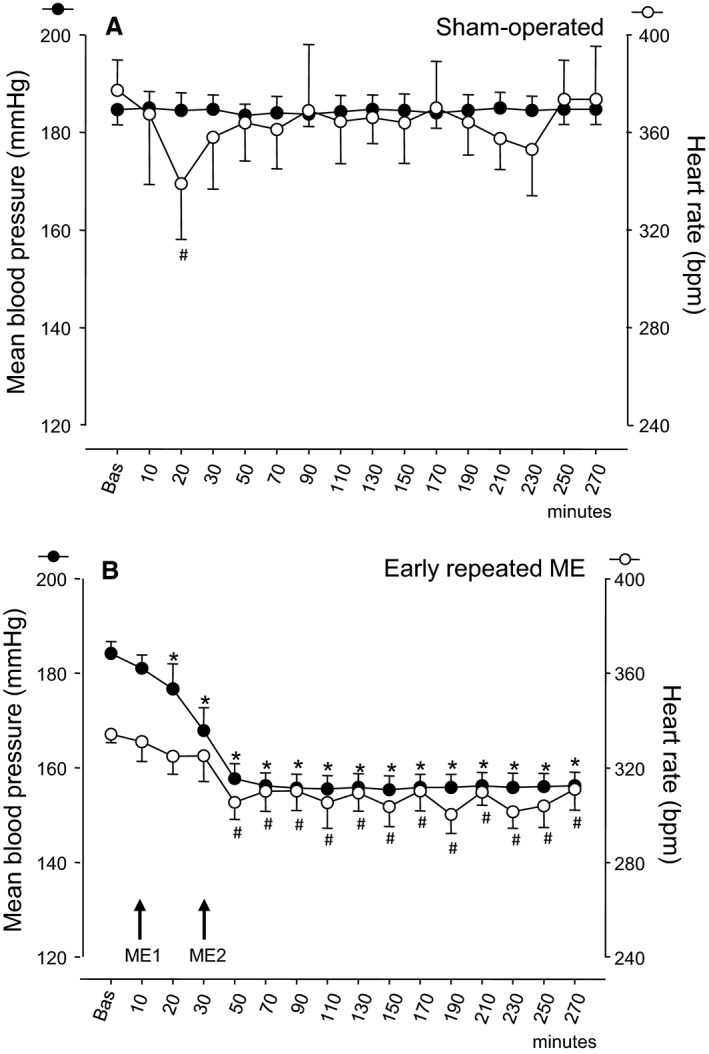
The time courses, in SHR, of the mean blood pressure (left ordinate axis, filled circles) and heart rate (right ordinate axis, empty circles) in (A) sham‐operated rats and (B) early repeated ME treated rats. ME1 and ME2 with arrows indicate the timing of ME treatments, and “Bas” indicates the mean basal value. Values (means ± SEM) are plotted every 10 min for the first 30 min after basal values and every 20 min, thereafter. Asterisks (*) and hashes (#) indicate significant differences in post hoc comparisons between basal value and posttreatment values, respectively for mean blood pressure and heart rate. For other explanations see text.

The time course of MABP and HR in the two study groups is shown in Figure 4. Post hoc comparisons are also reported, if significant.

For MABP, no significant change was present in the sham‐operated group for the entire observation period (Fig. 4A).

In rats with early repeated ME (Fig. 4B), MABP displayed a marked decrease of about 30 mmHg 20 min after ME2 and remained then stably low for the subsequent observation period of about 4 h. Overall repeated measure ANOVA revealed a highly significant main effect (*P* < 0.001) and post hoc comparisons against the baseline value were significant for all observation points after 20 min.

For HR, ANOVA revealed a weakly significant main effect of treatment (*P* = 0.040) in sham‐operated rats and a highly significant main effect (*P* < 0.001) in early repeated ME. Post hoc comparisons revealed a single significant value lesser than baseline (Fig. 4A), whereas for early repeated ME all values from 50 min onwards were significantly lesser than baseline (Fig. 4B).

## Discussion

In this paper, we have compared the effect of mandibular extension in two models of experimental hypertension in the rat.

Dexamethasone‐induced hypertension (Dex‐HT) is a model of glucocorticoid‐induced hypertension. The exact mechanism of blood pressure elevation is still unknown, and perturbations in various pathophysiological systems affecting blood pressure, have been proposed as contributing to Dex‐HT (Ong et al. 2009), including oxidative stress (Zhang et al. 2004; Ong et al. 2007). SHR is a well‐established and widely studied model of spontaneous hypertension in the rat (Ong et al. 2009).

This study provides confirmatory results of previous studies in normotensive rats. In fact, (1) after sham‐operation, MABP and HR remained stable over the prolonged (up to 4 h) observation period; (2) MABP dropped after a single ME for about 2 h by about 20 mmHg; and (3) ME repetition markedly prolonged the hypotensive effect (i.e., for the entire observation period). In addition, and this is a new finding, in Dex‐HT rats and SHR the hypotensive response to repeated ME was found to be greatly enhanced in magnitude compared both to single ME (in normotensive and hypertensive rats) and to repeated ME in normotensive rats, that is, by about 40 mmHg.

The response of HR to the various ME applications is less straightforward than that of MABP. In all instances of ME application (but not in sham conditions), HR tended to significantly decline after ME, even if not always paralleling the time course of MABP.

It may be interesting to note that, since the decline involved both HR and MABP, an even more marked reduction must have resulted for the so‐called rate pressure product, which represents a useful estimator of cardiac workload. Unfortunately, the precise calculation of cardiac load reduction cannot be done on these data, since it requires the determination of systolic blood pressure, rather than mean blood pressure, as available in this study.

Being the MABP lowering response to mandibular extension a seemingly novel phenomenon, only a few aspects have been clarified, regarding its mechanism.

In the normotensive rat, this effect was found to be abolished by bilateral peripheral trigeminal section (Lapi et al. 2013), suggesting a role of this cranial nerve as the afferent limb of the response. The role of trigeminal nerves is also indicated by the studies of Kumada and Schaller, who observed, respectively in neurophysiological studies in the anesthetized rabbit (Kumada et al. 1977) and in humans during cranio‐facial surgery (Schaller 2004; Schaller et al. 2008, 2009; Chowdhury et al. 2015) that trigeminal nerve stimulation are followed by (albeit brief) hypotensive and bradycardic responses. The responses of the cardiovascular system to trigeminal stimulation are also referred to as trigemino‐cardiac reflexes and have been extensively described by Schaller and co‐workers (Meuwly et al. 2015a,b).

Furthermore, studies by us on normotensive rats have demonstrated that mandibular extension, in addition to reduce blood pressure, also determines a prolonged arteriolar pial vasodilation (Lapi et al. 2013, 2014, 2017). This effect is apparently mediated by a local activation of the nitric oxide system, as suggested by the fact that mandibular extension‐induced arteriolar pial vasodilation was abolished by administering an inhibitor of nitric oxide synthase (L‐NAME) (Lapi et al. 2014). Interestingly, the time course of the pial arteriolar response was similar to that of MABP, both after a single ME (full duration = 2 h) and after early repeated ME (at least 4 h) (Lapi et al. 2017).

We have previously proposed that the hypotensive response to mandibular extension may be related to the so‐called postexercise hypotension (PEH), that is, the sustained reduction in arterial blood pressure that is observed after muscular exercise (Kenney and Seals 1993; MacDonald 2002). In fact, single bouts of various types of exercises, including dynamic aerobic exercise or dynamic resistance exercise or isometric resistance exercise (Kenney and Seals 1993; Pescatello and Kulikowich 2010; Brook et al. 2013, 2015; Kruse et al. 2016; Ash et al. 2017) and passive skeletal muscular stretching (Wong and Figueroa 2014; Kruse et al. 2016) have been demonstrated to be followed by a prolonged blood pressure reduction in the human. Evidence of PEH has been provided also for the rat (Borges et al. 2014).

Although we were not able to find studies on the effect of single repeated exercise on blood pressure either in the rat or in the human, there is ample evidence that multiple repeated brief aerobic exercises interspersed throughout the day result in a stable reduction of blood pressure, as discussed by Pescatello (2015).

A few limitations of the study should be acknowledged. Firstly, we would note that studies were done on rats anesthetized for 5–8 h, and that anesthesia, especially when prolonged, could have contributed to the response of MABP. On the other hand, we should note that in sham‐operated (control) anesthetized rats MABP and HR remained stable for the entire observation period of at least 5 h. A further point is the essentially descriptive nature of this study that requires further investigations on the underlying mechanisms. Nonetheless, we feel that the magnitude and the length of the observed phenomenon deserved to be reported. Further studies in conscious rats and in humans are needed to assess whether this or similar procedures could find an application as a non‐pharmacological ancillary aid in acute or chronic treatment of arterial hypertension.

## Conflict of Interest

The authors have no conflicts of interest to declare.
